# DNA methylation mediated silencing of microRNA-874 is a promising diagnosis and prognostic marker in breast cancer

**DOI:** 10.18632/oncotarget.17569

**Published:** 2017-05-02

**Authors:** Lei Zhang, Da-Li Yan, Fan Yang, Dan-Dan Wang, Xiu Chen, Jian-Zhong Wu, Jin-Hai Tang, Wen-Jie Xia

**Affiliations:** ^1^ Graduate School, Xuzhou Medical University, Xuzhou, Jiangsu, P.R. China; ^2^ Department of General Surgery, Nanjing Medical University Affiliated Cancer Hospital, Cancer Institute of Jiangsu Province, Nanjing, Jiangsu, P.R. China; ^3^ Department of General Surgery, Jiangsu Cancer Hospital Affiliated to Nanjing Medical University, Nanjing, Jiangsu, P.R. China; ^4^ Department of General Surgery, The First Affiliated Hospital with Nanjing Medical University, Nanjing, Jiangsu, P.R. China

**Keywords:** breast cancer, DNA methylation, miR-874, prognosis, TCGA

## Abstract

MicroRNA-874 (miR-874) is downregulated in several human cancers and has been suggested to be a tumor suppressor gene. However, the molecular mechanism of miR-874 downregulation in breast cancer has not been well elucidated. Here we aimed to study the aberrant hyper-methylation of CpG sites with the utility of miR-874 downreregulation in breast cancer and evaluate the clinical function of miR-874 as a prognostic marker. The miR-874 expressions in cells and tissues of two breast cancer lines were measured by real-time PCR. The DNA methylation status of the miR-874 promoter region in 19 pairs of breast cancer and adjacent normal samples was analyzed with Sequenom EpiTYPER MassArray. To evaluate whether miR-874 is a potential prognostic marker in breast cancer, we also explored the clinical long-time follow-up records from The Cancer Genome Atlas (TCGA). We found miR-874 expression was downregulated in 47 pairs of breast cancer tissues. Moreover, univariate and multivariate analysis revealed miR-874 expression may be a prognostic biomarker of overall survival in breast cancer patients. Preconditioning with 5-Aza-CdR in two cell lines elevated miR-874 expressions. The data from Sequenom EpiTYPER MassArray showed that DNA methylation of the promoter region of miR-874 was upregulated and accompanied by decreased miR-874 expression, which was further confirmed by TCGA. After comprehensive considerations, we think miR-874, which might be served as a prognostic biomarker, is mediated by DNA methylation.

## INTRODUCTION

Breast cancer is one of the most commonly diagnosed cancers and a major cause of cancer-related death in women worldwide [1]. The existing screening techniques and treatment methods (including neoadjuvant chemotherapy, surgical methods, cytotoxic chemotherapy, radiotherapy, and targeting agents against hormone receptors and HER-2) have still low prognostic values for breast cancer [2, 3]. Thus, it is important to identify more reliable prognostic markers that are effective in prevention and treatment of breast cancer.

MicroRNAs (miRNAs) are short highly-conserved small non-coding RNA molecules of 19 to 25 nucleotides that regulate post-transcriptional gene expressions. By targeting complementary binding sites within the 3′-untranslated region (3′-UTR) of target messenger RNAs, they impair or inhibit translation and promote degradation [4–6]. MiRNAs play important roles in gene expression regulation and control diverse physiological and pathological processes. miRNAs are involved in regulating various bioprocesses, including apoptosis, proliferation, cell differentiation, metabolism, signal transduction and carcinogenesis [7, 8].

Recently, several reports show that miRNA-874 (miR-874) expression is downregulated and functions as a tumor suppressor in several cancers, including non-small cell lung cancer (NSCLC) [9], breast cancer [10], osteosarcoma [11, 12], gastric cancer [13, 14], colorectal cancer [15], testis cancer [16], and maxillary sinus squamous cell carcinoma [17]. Moreover, miR-874 is significantly downregulated in breast cancer cells and its overexpression could suppress growth, colony formation and cycle of breast cancer cells and increase cell apoptosis [10]. miR-874 is also involved in tumor progression by suppressing the protein expressions of matrix metalloproteinase-2 (MMP-2) and uPA and targeting CDK9, E2F3, HDAC1, aquaporin-3, HCA587/MAGE-C2 and signal transducer-activator of transcription 3 (STAT3), a key transcription factor that plays a significant role in human cancer angiogenesis [9–16, 18, 19]. Therefore, only downstream mechanisms of miR-874 altering cellular processes are confirmed. Here we aim to investigate the upstream molecular mechanism of down-regulated miR-874 expression in breast cancer.

Accumulating evidences demonstrate that the dysregulation of miRNA subsets is attributed to genetic and epigenetic alterations [20, 21]. Among them, DNA methylation of CpG islands in the promoter region of miRNAs (e.g. miR-145, -497, -874, -148a and -34b [15, 22–26], plays critical roles in chromatin remodeling and general gene expression regulation during mammalian development and tumorigenesis [27, 28]. Here to prove our notion, we tested two breast cancer cell lines and freshly resected breast cancer tissues. To our knowledge, this is the first study to investigate the upstream molecular mechanism of down-regulated miR-874 expression in breast cancer.

It is demonstrated that miR-874 expression is correlated with tumor size, tumor/node/metastasis (TNM) staging and lymph node metastasis [13]. Given its critical role in tumor development and progression, we also explored the utility of miR-874 as a prognostic marker by integrating miR-874 and DNA methylation next-generation sequencing data from The Cancer Genome Atlas (TCGA), which was proved convictive and appropriate by previous molecular studies. We applied for 1089 primary invasive ductal carcinomas (IDCs) in the TCGA cohort, the most frequent type of breast cancer, and used long-time follow-up records to evaluate the correlations of miR-874 expression with clinic-pathological parameters and overall survival (OS) in breast cancer patients. The findings suggest that miR-874, which is mediated by DNA methylation, might serve as a prognostic biomarker in breast cancer patients.

## RESULTS

### miR-874 downregulation is correlated with poor prognosis of breast cancer patients

**Figure 1:**
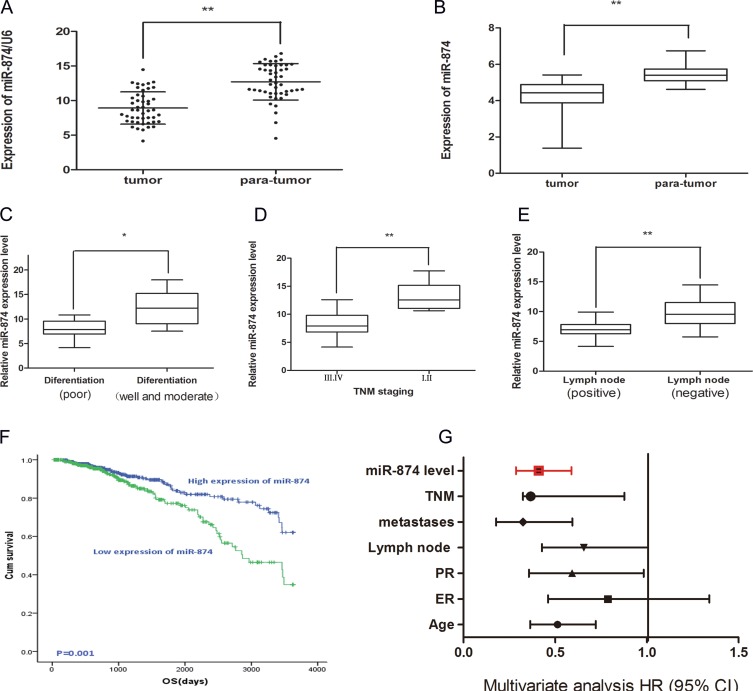
(**A**) Real time-PCR demonstrated miR-874 expression in 47 patients with breast cancer compared with matched para-normal tissues. Symbol (**) means a significant difference at *P* < 0.01. (**B**) The expression of miR-874 in breast cancer tissues compared with para-normal tissues from external breast cancer cohort in TCGA database. (**C**) miR-874 expression in moderately and well differentiation tissues as well as tissues with poor differentiation. (**D**) miR-874 expression in I of TNM staging compared with II or III of TNM staging. (**E**) miR-874 expression in negative lymph node metastasis tissues compared to positive lymph node metastasis tissues. (**F**) The high and low miR-874 expression groups of OS were depicted using the Kaplan-Meier. (**G**) The multivariate analysis of the HRs by Cox multivariate proportional hazard regression model.

To explore whether miR-874 was associated with several clinic-pathologic parameters, we detected the miR-874 expressions in 47 patients without receiving neoadjuvant therapy. Quantitative real-time polymerase chain reaction (qRT-PCR) shows that miR-874 expressions are downregulated in 47 pairs of breast cancer samples and matched paranormal tissues (Figure 1A). The miR-874 expressions are also downregulated in breast cancer tissues compared with paranormal tissues from an external breast cancer cohort in TCGA (Figure 1B), indicating that miR-874 is significantly down-regulated in breast cancer (*P* < 0.01). Accordingly, we used the median miR-874 expression as a cutoff point and divided the 47 breast cancer patients into a high-expression group (*n* = 24) and a low-expression group (*n* = 23). Furthermore, we find miR-874 expression is associated with pathological differentiation, TNM staging and lymph node metastasis in breast cancer tissues. As depicted in Figure 1C–1E, miR-874 expression is downregulated in breast cancer tissues with poor differentiation, TNM staging III and IVand positive lymph node examined by *t*-test (all *P* < 0.05). Considering the small sample size and some samples without long-time follow-up records, we also applied the clinical parameters with long-time follow-up records to investigate the association between miR-874 expression and OS from an external breast cancer cohort in TCGA (*n* = 1089). As depicted in Table 1, the miR-874 expression is correlated with the status of estrogen receptor (ER), TNM stage and lymph node metastasis calculated by the same methods (all *P* < 0.05), which proves the database is convictive and appropriate compared to the above results. Moreover, patients with high miR-874 expression exhibit better OS (HR = 0.425, *P* = 0.001; Figure 1F). The univariate and multivariate analysis further indicates the miR-874 expression may be a significant prognostic biomarker in breast cancer patients (*P* < 0.01) (Table 2 and Figure 1G). Collectively, these results suggest that miR-874 expression might serve as a novel prognostic biomarker in breast cancer.

**Table 1:** Correlation between miR-874 expression and different clinicopathological features in breast cancer

Variable	Cases (*n*)	Expression level of miR-874		*P*-value
		Low (*n*, %)	High (*n*, %)	
**Ages**				
< 60	590	291	299	0.603
≥ 60	499	254	245	
**ER**				
positive	842	435	407	0.049*
negative	247	110	137	
**PR**				
positive	737	368	369	0.913
negative	435	177	175	
**Lymph node**				
negative	504	252	252	0.001*
1∼3	386	179	207	
4∼9	123	56	67	
≥ 10	76	58	18	
**Distant metastasis**				
No	1066	531	535	0.294
Yes	23	14	9	
**Tumor sizes**				
< 2 cm	291	132	159	0.062
≥ 2 cm	798	13	385	
TNM staging				
**I.II**	819	392	427	0.012*
**III IV**	270	153	117	

^*^P < 0.05 calculated with Pearson chi^2^ test.

**Table 2:** Univariate and multivariate Cox regression analyses of overall survival in breast cancer patients

Univariate analysis				Multivariable analysis		
Parameters	HR	95%CI	*P* value	HR	95%CI	*P* value
Age (> 60)	0.587	0.421–0.818	0.002*	0.513	0.364–0.721	0.001*
ER (negative)	0.645	0.453–0.919	0.015*	0.786	0.461–1.340	0.376
PR (negative)	0.680	0.486–0.951	0.024	0.592	0.357–0.983	0.043
Lymph node metastasis (positive)	0.471	0.329–0.674	0.001*	0.656	0.428–1.006	0.053
Tumor sizes (> 2 cm)	0.681	0.461–1.006	0.053	-	-	-
Distant metastases (positive)	0.229	0.134–0.393	0.001*	0.325	0.178–0.594	0.001*
TNM (III and IV)	0.450	0.321–0.630	0.001*	0.567	0.366–0.877	0.011*
miR-874 level (low)	0.425	0.298–0.606	0.001*	0.410	0.286–0.589	0.001*

### DNA methylation is responsible for miR-874 downregulation from TCGA

**Figure 2:**
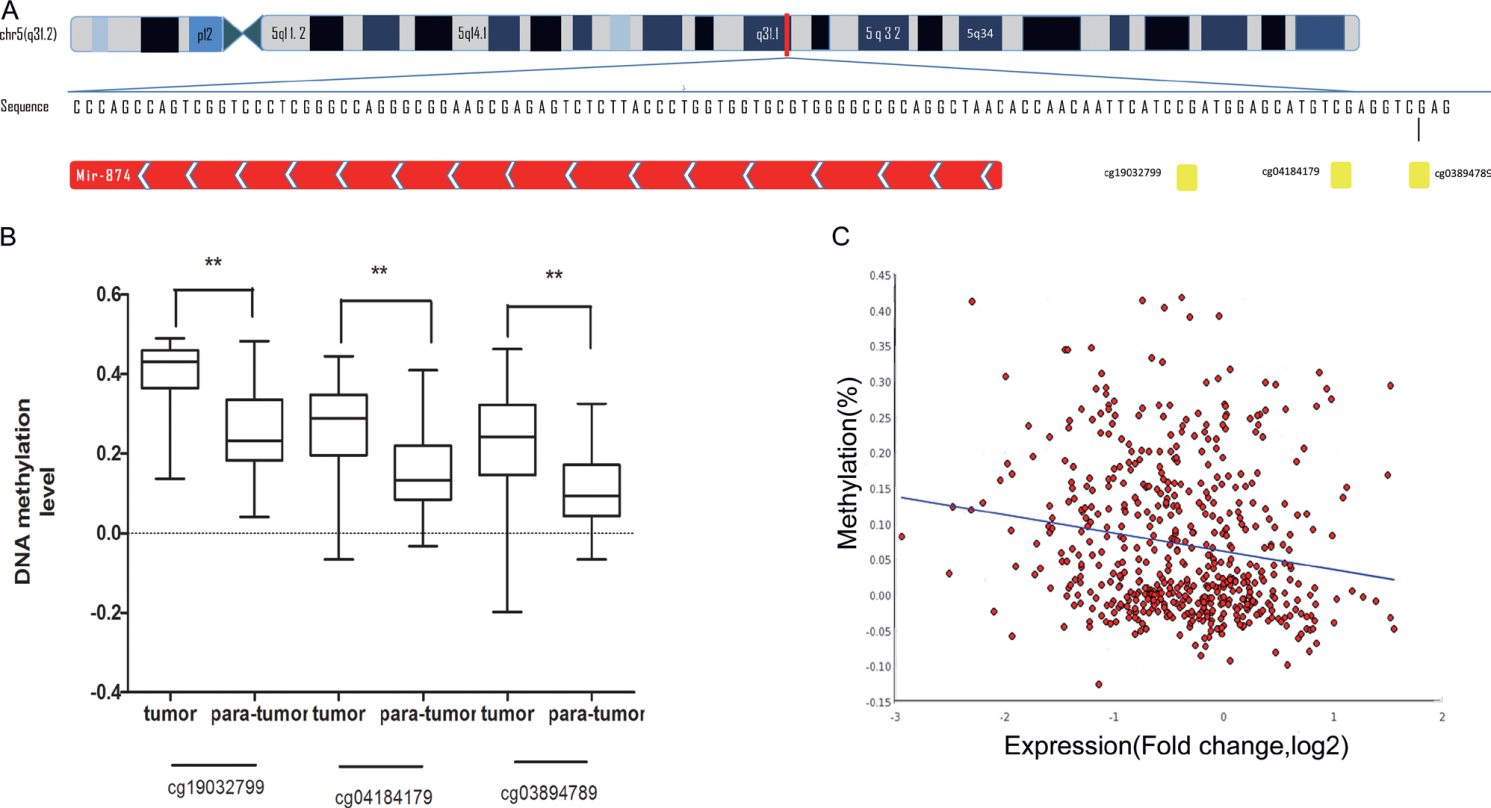
(**A**) Schematic representation of the location of miR-874 in chr5:136983261-136983338. Three CpG sites(cg19032799, cg04184179, cg03894789 ), located on chromosome chr5:136983354, chr5: 136983367 and chr5: 136983373, represented as yellow spot located in the promoter region of miR-874. (**B**) The methylation level for three CpG sites (cg19032799, cg04184179, cg03894789) in breast cancer tissues compared with para-normal tissues from external breast cancer cohort in TCGA database. (**C**) miR-874 expression negatively correlated with against the DNA methylation levels of its 300-upstream region (spearmanr = −0.321, *P* value < 0.01) depicted MethHC (http://MethHC.mbc.nctu.edu.tw).

In consideration of its critical role in clinic-pathologic parameters and prognosis, we tried to apply the newly available TCGA to validate the relationship between miR-874 expressions and DNA methylation and determine the mechanism of miR-874 down-regulation. As shown in Figure 2A, miR-874 is located on chromosome chr5: 136983261-136983338. TCGA includes three CpG sites of methylation levels in the 5′end of miR-874 (cg19032799, cg04184179, cg03894789), which are a part of the 8 CpG sites measured in our study. The data downloaded from Cancer Browser (https://genome-cancer.ucsc.edu/) show that the DNA methylation levels in the miR-874 promoter region are remarkably elevated in breast cancer tissues compared to paratumor tissues (Figure 2B). Data from MethHC (http://MethHC.mbc.nctu.edu.tw) show a significant negative correlation between miR-874 expression and methylation levels in its 300-upstream region (Figure 2C). Further analysis of 873 breast cancer with DNA methylation levels intensely suggests that DNA methylation may be a common mechanism of miR-874 expression silencing in breast cancer.

### miR-874 downregulation is due to aberrant CpG methylation of miR-874 gene promoter region in breast cancer

**Figure 3:**
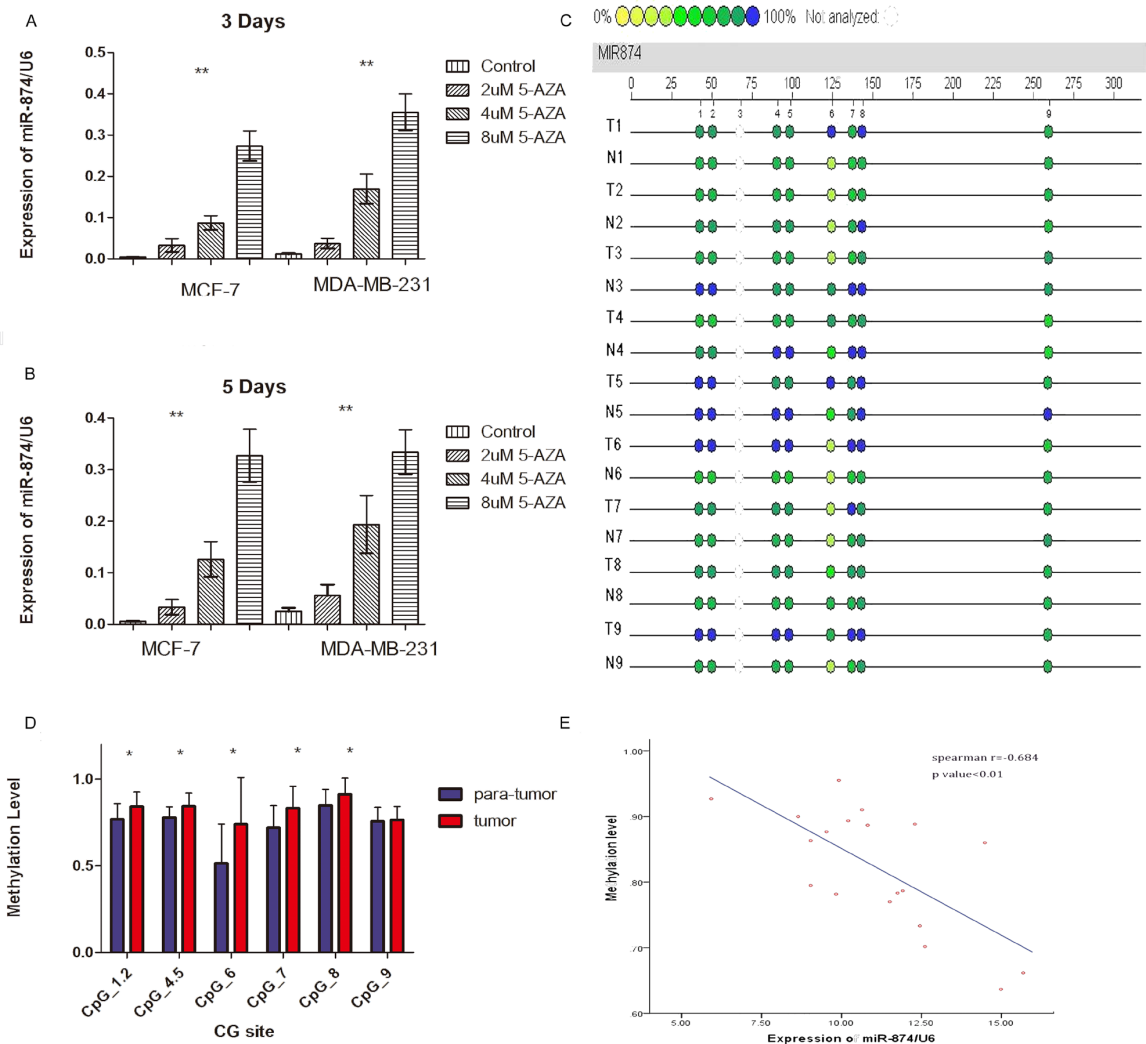
(**A**, **B**) Real time-PCR demonstrated miR-874 expression in two breast cancer cell lines after treatment with different proportions of 5-aza-2-deoxycytidine for 3 or 5 days compared with control cells. Symbol (**) means a significant difference at *P* < 0.01. (**C**) Profiling of the methylation level of CpG sites in the miR-874 promoter region. (**D**) Average methylation level of each CpG site in para-tumor tissues and breast cancer tissues. Symbol (*) means a significant difference at *P* < 0.05. (**E**) Correlation analysis between average methylation levels of the promoter region and relevant miR-874 expressions.

**Figure 4:**
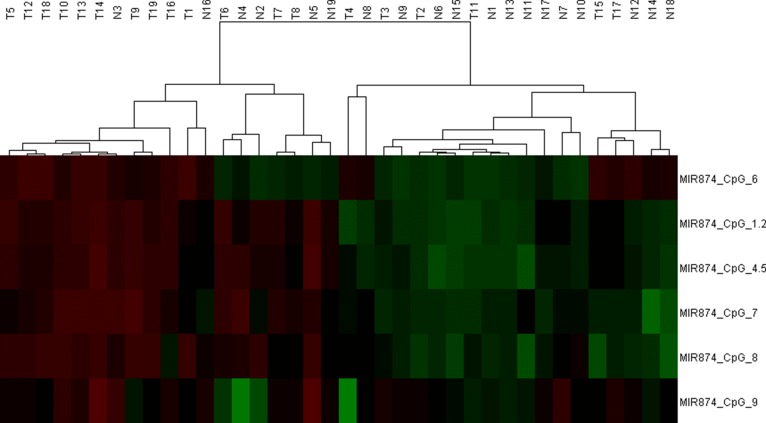
We performed an unsupervised two-dimensional hierarchical clustering analysis, which provides an unbiased view on these relationships DNA-methylation values are shown in this false-color image on a continuous scale from green ( 0% methylated) to red (100% methylated).

To further explore the upstream molecular mechanism of down-regulated miR-874 expression in breast cancer, we assessed the DNA methylation status of the miR-874 promoter region in both breast cancer cells and tissues. As depicted in Figure 3A, 3B, the 3- or 5-day inhibition of DNA methylation with different 5-aza-CdR concentrations significantly increased miR-874 expression in two breast cancer cell lines (MCF-7 and MDA-MB-231), suggesting a significant negative correlation between miR-874 expression and methylation levels. In addition, DNA methylation level of 8 CpG sites at upstream of miR-874 gene were measured in 19 paired breast cancer and their matched para-tumor samples by using Sequenom EpiTYPER MassArray, a bisulfite-treatment-based method for detection and quantitation of DNA methylation (Figure 3C, 3D and Table 3). We also performed an unsupervised two-dimensional hierarchical clustering, which provides an unbiased view on these relationships (Figure 4). Results indicate 7 of the 8 CpG sites located at the upstream of miR-874 are hyper-methylated in tumors compared with the matched para-tumor tissues. Then we plotted the correlation between miR-874 expression (x-axis) and mean methylation level (y-axis) in breast cancer (Figure 3E) and found a significant reverse correlation (Spearman *r* = −0.684, *p* < 0.01). Comprehensively, these data suggest that DNA hyper-methylation in the upstream region of miR-874 might play a significant role in miR-874 expression downregulation in breast cancer.

**Table 3:** Comparison of methylation level between breast cancer and adjacent normal tissue in each CpG site

CpG sites	Number of pairs	Tissue types	Mean methylation level ± SD	Paired *t* value
CpG1,2	19	para-tumor breast cancer tissue	0.7684 ± 0.090 0.8421 ± 0.083	2.607*(0.013)
CpG4,5	19	para-tumor breast cancer tissue	0. 7784 ± 0.062 0. 8442 ± 0.075	2.939*(0.006)
CpG6	19	para-tumor breast cancer tissue	0.5142 ± 0.226 0.7400 ± 0.268	2.805*(0.008)
CpG7	19	para-tumor breast cancer tissue	0.7205 ± 0.127 0.8232 ± 0.125	2.504*(0.017)
CpG8	19	para-tumor breast cancer tissue	0.8495 ± 0.091 0.9137 ± 0.092	2.166*(0.037)
CpG9	19	para-tumor breast cancer tissue	0.7568 ± 0.080 0.7663 ± 0.0756	0.375(0.710)

## DISCUSSION

Increasing evidence indicates that miRNAs could act as oncogenes or tumor suppressors and are involved in both biological and pathological processes in several cancers, including proliferation, angiogenesis and metastasisI [29, 30]. Moreover, miRNAs play essential roles in tumor development and progression, and several tumor suppressive miRNAs are associated with clinical outcomes in breast cancer [31]. Thus, exploring the mechanism and role of miRNAs in breast cancer is significant for its diagnosis and therapy. miR-874 is located on chromosome 5q31.2, a well-known frequent fragile site where the human genome is specifically correlated with chromosomal rearrangements in cancers [32]. As reported, miR-874 is downregulated in NSCLC, breast cancer, osteosarcoma, gastric cancer, colorectal cancer, and maxillary sinus squamous cell carcinoma. In addition, miR-874 is involved in tumor progression by suppressing the protein levels of MMP-2 and uPA and targeting E2F3, HDAC1, AQP3 STAT3 and HCA587/MAGE-C2 in a variety of cancers [10–16, 18, 33]. Furthermore, miR-874 may function as a competing endogenous RNA to regulate the AQP3 expression through competition with lncRNA H19, which has received much attention for years [19]. As reported, miR-874 overexpression in breast cancer cells can suppress cell growth and increase cell apoptosis, while miR-874 overexpression could dramatically suppress tumorigenicity in nude mice *in vivo* [10]. Therefore, we determined the miR-874 expressions in breast cancer tissues and matched normal tissues using RT-PCR, which revealed a drastic decrease of miR-874 expression in the breast cancer tissues. Importantly, the novel finding in our study is that miR-874 level downregulation is correlated with lymph node metastasis and TNM staging in breast cancer. Moreover, Kaplan-Meier survival analysis and multivariate analysis further indicate that miR-874 might be regarded as a novel potential prognostic maker from an external breast cancer cohort in TCGA.

As reported, miR-874 expression is remarkably down-regulated and miR-874 may function as an oncogenic or tumor suppressor in various malignancies. Several studies partially substantiated the downstream mechanisms of miR-874 altering cell processes. However, the upstream mechanism underlying how miR-874 is down- regulated in breast cancer still has not been well elucidated. Recent studies suggest many tumor-suppressor miRNAs are downregulated due to promoter hyper- methylation of the CpG islands in several cancers [34, 35]. To explore the epigenetic mechanism mediating miR-874 in breast cancer, we detected miR-874 expression after de-methylation treatment or pretreatment with 5-aza-CdR in two breast cancer cell lines and found it was remarkably upregulated. This result suggests hyper- methylation plays a significant role in regulation of miR-874 expression in breast cancer. We also evaluated the DNA methylation levels of 8 CpG sites at the upstream of miR-874 in 19 paired breast cancer and matched normal tissues by using MassARRAY-based quantitative methylation analysis. Results show miR-874 expression is downregulated in breast cancer tissues in association with elevated DNA methylation, which is further confirmed in a large cohort from TCGA. To our knowledge, this is the first investigation about the aberrant miR-874 expression due to DNA methylation in breast cancer.

After comprehensive considerations, we think miR-874, which might serve as a prognostic biomarker or potential therapeutic strategy, is mediated by DNA methylation in breast cancer patients.

## MATERIALS AND METHODS

### Specimens

Breast cancer specimens and their matched normal breast tissues were collected from patients treated at Jiangsu Cancer Hospital, Affiliated Hospital of Nanjing Medical University between 2014 and 2016. The specimens were snap- frozen in liquid nitrogen. The study protocol was approved by the Ethics Committee of Jiangsu Cancer Hospital, and informed consent was obtained from all patients.

### Cell culture and treatment with 5-aza-2′deoxycytidine (5-Aza-CdR)

Human breast cancer cell lines MCF-7 and MDA-MB-231 were purchased from American Type Culture Collection (ATCC, USA). Both cell lines were cultured in the Roswell Park Memorial Institute 1640 medium (RPMI-1640, Keygen, Nanjing China) supplemented with 10% fetal bovine serum (Gibco, Life Technology) at 37°C in a humidified atmosphere with 5% CO_2_. After 24 hours of serum starvation, the two cell lines were separately seeded in six-well plates, allowed to attach for 3 or 5 days and treated with 0, 5 or 10 μM 5-Aza-CdR.

### Total RNA extraction, reverse transcription and qRT-PCR

Total RNA was extracted using an RNAsimple Total RNA kit (Tiangen Biotech, Beijing, China) according to the manufacturer's instructions. The concentration and quality of the RNA were measured on a Nanodrop 2000 spectrophotometer (Thermo Scientific, USA) at the ultraviolet wavelengths of 260 and 280 nm (260/280 nm). Mature miR-874 levels were quantified using TaqMan microRNA Assay (Applied Biosystems). Expression of miRNA was analyzed using a MiR-X miRNA qRT-PCR SYBR Kit (638314; Clontech Laboratories, USA) on Roche LightCycler 480 II, with the manufacturer-provided mRQ 3′ Primer and miRNA-specific forward primer. The miRNA expressions in cells and tissues were normalized using U6 snRNA as an endogenous control. The cycle threshold (Ct) of each gene was normalized to the internal control, and the relative fold change was calculated using the ΔΔCt method in triplicate.

### TCGA validation

Data were downloaded from the TCGA portal and processed partly with MethHC from Institute of Bioinformatics and Systems Biology at National Chiao Tung University, Hsinchu, Taiwan (http://MethHC.mbc.nctu.edu.tw). The discriminating methylation levels of the three CpG sites (cg19032799, cg04184179, cg03894789), the miR-874 expression and clinic-pathologic data in breast cancer and non-tumor tissues were analyzed on basis of the gene chip data (TCGA_BRCA_ hMethyl450) and (TCGA_BRCA_miRNA) from Cancer Browser (https://genome-cancer.ucsc.edu/).

### DNA extraction and methylation analyses

Genomic DNAs were extracted from 26 paired breast cancer specimens and matched normal tissues using a genomic DNA rapid extraction kit (Tiangen Biotech Co, Ltd). The quality and quantity of DNA were evaluated by gel electrophoresis and a NanoDrop spectrophotometer (GE Healthcare Life Sciences, Uppsala, Sweden), respectively, but 7 of the 26 paired tissues were not qualified. Genomic DNA from each specimen was isolated using a QIAamp DNA Mini Kit (Qiagen) and modified by bisulfite using an Epitect Bisulfite Kit (Qiagen) according to the manufacturer's instructions. Methylation of the miR-874 promoter was quantitatively analyzed on Sequenom MassARRAY (CapitalBio), which employs matrix-assisted laser-desorption/ionization time-of-flight mass spectrometry and RNA base-specific cleavage. Pairs of primers on basis of the reverse complemented strand of miR-874 (GTG AGTTGGGTAGAAGG TAGGTTTA, TATATCTAATTCC AAAACCCATTTTT) were used to amplify base pairs -193–(+29) around miR-874. Each forward primer was added with a 10-mer tag (5′-AGG AAGAGAG-3′) and each reverse primer with a T7-promoter tag (5-CAGTAATAC GACTCACTATAG GGAGAAGGCT-3′) for transcription *in vitro*. Altogether, 8 CpG sites in this region were checked. The spectra methylation ratios were generated on Epityper 1.0 (Sequenom, San Diego, CA).

### Statistical analysis

All statistical analyses were performed using SPSS version 19.0 software (SPSS Inc., Chicago, USA). Student's *t*-test and paired *t*-test were applied to evaluate methylation level of miR-874. A linear regression was performed to infer the correlation between the methylation level and expression of miR-874. The connection between miR-874 expressions and clinical parameters were analyzed by the chi^2^ test. Survival curves were exhibited using the Kaplan–Meier method, the log-rank test and Cox's proportional. Statistical significance was set at *P* < 0.05.
